# Temporal Changes in Plasma Concentration of Leptin, IGF-1, Insulin and Metabolites Under Extended Fasting and Re-Feeding Conditions in Growing Lambs

**DOI:** 10.5812/ijem.6529

**Published:** 2012-12-21

**Authors:** Ali Kiani

**Affiliations:** 1 Animal Sciences Group, Lorestan University, Khoramabad, IR Iran

**Keywords:** Leptin, Insulin-Like Growth Factor I, Energy Metabolism, Sheep

## Abstract

**Background:**

A fall in plasma concentration of energy status related hormones (leptin, insulin-like growth factor-1 (IGF-1) and insulin) and body energy expenditure occurs in response to short term fasting. Nevertheless, the relations of the fasting-induced changes in energy related hormones and metabolites with fasting energy expenditure (FEE) under extended fasting condition have received little attention so far.

**Objectives:**

It is not clear how energy status related hormones coordinate to cope with feed deprivation under extended fasting time conditions and how quickly these hormones re-bound to fed-state values in response to re-feeding. Thus the objectives of this study were: 1) to determine the effects of extended fasting on plasma concentration of leptin, IGF-1, insulin, glucose, NEFA, 3-β-hydroxybutyrate (BOHB) and urea; and 2) to study the relations of energy status related hormones with FEE and substrate oxidations under extended fasting conditions.

**Materials and Methods:**

Eighteen six-month-old growing lambs (9 females and 9 males) were fasted for three days. Blood samples were taken one hour before (-1H) and 48 and 72 hours after fasting (48H and 72H) and two hours after re-feeding (+2H) from jugular vein. During the last 22 hours of fasting, gas exchange (CO2 production and O2 consumption) were measured using an open-circuit indirect calorimeter. Respiratory quotient (RQ), FEE and relative proportions of oxidized protein, fat and carbohydrate were calculated.

**Results:**

Plasma levels of leptin, insulin, IGF-1 and glucose decreased but NEFA and urea levels increased within 48H of fasting. Concentration of insulin significantly increased with extended fasting while leptin and IGF-1 levels remained constant. Glucose was the only blood variable that showed a quick re-bound within two hours after re-feeding. Leptin and IGF-1 showed significant positive relations with glucose and BOHB but negative relations with NEFA and Urea. Carbohydrate, fat and proteins contributed to 17%, 61% and 22% of FEE respectively in three-day-fasted lambs. FEE was negatively correlated with insulin and NEFA concentrations in plasma.

**Conclusions:**

Even though plasma levels of leptin and IGF-1 decreased and remained constant under extended fasting, neither leptin nor IGF1 re-bounded to fed-status values within two hours after re-feeding. Under extended fasting condition, firstly an insulin resistance develops and secondly, a fall in FEE through a switch from carbohydrate- to fat-based metabolism occurs and there is an evident negative correlation between FEE and plasma concentration of NEFA.

## 1. Background

During feed-deprivation or fasting, different metabolic strategies coordinate in favor of vital organs (such as brain). Plasma levels of different hormones and metabolites change temporally, consequently, stimulating gluconeogenesis and fat breakdown (1). On the other hand, the whole body energy expenditure reaches the lowest level in fasted animals. Nevertheless, the relations of fasting-induced changes in plasma hormones and metabolites with fasting energy expenditure have received little attention so far. Short-term fasting causes a fall in plasma concentration of energy status related hormones such as insulin and insulin-like growth factor-1 (IGF-1) and leptin. Among energy related hormones, leptin is an adipose-tissue derived satiety hormone that plays an important role in regulating food intake energy homeostasis in humans and in animals (2, 3). In fact, primary function of leptin is to signal body energy statues to the hypothalamus. Feed deprivation (chronic and acute reduced food intake) generally causes a reduction in plasma concentration of leptin. Low plasma leptin levels trigger a series of adaptive neuro-endocrine responses to increase food intake (2, 4, 5).

## 2. Objectives

In spite of a great body of information about the effects of short-term fasting on plasma concentration of energy status related hormones and metabolite, it is not clear how the body orchestrates the hormonal patterns to cope with feed deprivation under extended fasting time conditions and how quickly energy status related hormones re-bound to fed-state values in response to re-feeding. Thus the objectives of this study were: 1) to determine the effects of extended fasting on plasma concentration of leptin, IGF-1, insulin, glucose, NEFA, 3-β-hydroxybutyrate (BOHB) and urea; and 2) to study the relations of energy related hormones with fating energy expenditure (FEE) and substrate oxidations under extended fasting conditions in growing lambs.

## 3. Materials and Methods

### 3.1. Animals and Experimental Protocols

All experimental procedures complied with the related guidelines of and were approved by the National Committee on Animal Experimentation, Denmark. Eighteen growing lambs (9 females and 9 males) at the age of six months were fasted for 72 hours with no food and free access to water. The lambs were individually placed inside metabolic cages (59 cm wide, 160cm long and 80cm high) and kept at 10–15 ºC temperature , and 65–75 % humidity with 12-hour light–dark cycle throughout the fasting period.

### 3.2. Blood Sampling and Assays

Blood samples were collected one hour before (-1H) and 48 and 72 hours after fasting (48H and 72H) and two hours after re-feeding (+2H) at about 9:00 a.m. Blood samples were taken through jugular vein puncture, and collected in 10-ml heparin-flourine vacuum tubes (VacuntainerTM, Becton Dickinson Vacuntainer System Europe, Meylan Cradex, France) and 10-ml EDTA vacuum tubes (BD Vacuntainer System, Preanalytical Solution Deliver Industrial Estate Plymouth PL6 7 BP, UK). Blood samples were immediately cooled on ice and then centrifuged at 3000 r.p.m. for 15 min at 4 ºC within 30 min after collection. Plasma samples were transferred to polystyrene tubes (Hounissen, Rossikov, Denmark) and frozen at -20 ºC pending analysis. Plasma concentrations of glucose were analyzed by commercially available spectrophotometric kits (17-25 InfinityTM, Sigma Diagnostic® Inc, P. O. Box 14508, St. Louis, MO 63178, USA). The intra- and inter-assay CV of the glucose assays were 2.1% and 2.7 % respectively. Plasma insulin concentration was determined by a sandwich-type time-resolved fluoroimmunoassay (DELFIA) as described below (6). The intra- and inter-assay CV of the insulin assays were 5.4% and 3.3%, respectively. Plasma concentrations of NEFA were analyzed by spectrophotometric kits (NEFA: cat # 999-75406-01 NEFA-C, Wako Chemicals GmBH, Nissanstr. 2, D-41468 Neuss, Germany). The intra- and inter-assay CV of the NEFA assays were 4.7 % and 2.2 % respectively. Plasma concentrations of BOHB were determined spectrophotometrically as an increase in absorbance at 340 nm due to the production of NADH, at slightly alkaline pH in the presence of β-hydroxybutyrate dehydrogenase (7). The intra- and inter-assay CV of the BOHB assays were 2.3% and 4% respectively. Plasma urea-N concentrations were determined enzymatically as described by Roch-Ramel F et al (8). Intra- and inter-assay CV of the urea assays were 2.0% and 2.0 % respectively. Plasma samples for leptin were freeze-dried and analyzed at the University of Western Australia, Perth. Leptin analyses were performed in duplicate by a double-antibody radioimmunoassay using ovine leptin raised against bovine leptin as described by Blache D et al (9). The limit of detection level was 0.07 ng/ml. Plasma IGF-I concentration was determined by double-antibody RIA with human recombinant IGF-I and antihuman IGF-I antiserum according to Breier BH et al (10). The intra- and inter-assay CV for leptin and IGF-I assays were below 5% and 10% respectively.

### 3.3. Fasting Energy Expenditure Measurements

An open-circuit indirect calorimeter (temperature 15–18 ºC, humidity 65–75 %, 12-hour light–dark cycle) was used. Lambs were kept inside respiration chambers during the last 22 hours of the 72 hours fasting period and gas exchanges were measured. Outgoing air was analyzed every three minutes for the concentration of O2 using a paramagnetic analyzer (Magnos 4G, Hartmann & Braun AG, Frankfurt, Germany), and for the concentrations of CO2 and CH4 using an infrared gas analysers (Uras 3, Hartmann & Braun AG) (11). Physical activity was not recorded in this experiment due to lack of proper instrument. Daily urine excretion was collected in 60 mL 10 % sulphuric acid and stored at –18 ºC for later chemical analyses. The nitrogen content in urine was determined by the Kjeldahl method using the Tecator-Kjeltec system 1026 (Tecator AB, Höganäs, Sweden) distilling unit. 

Fasting energy expenditure (FEE) was calculated from O2 consumption, CO2 and CH4 productions and urinary nitrogen excretion (UN) in accordance with Brouwer (12) as below: 

(1) FEE [kJ] = (16.18 [kJ/l] × O2 [l]) + (5.02 [kJ/l] × CO2 [l] ) – (2.17 [kJ/l] × CH4 [l]) – (5.99 [kJ/g] × UN [g]) 

Energy expenditure of oxidation of protein (OXP), carbohydrate (OXCHO) and fat (OXF) were calculated by the method described by Chewalibog et al. (13).

(2) OXP [kJ] = (UN [g] × 6.25) × 18.42[kJ/g]

(3) OXCHO [kJ] = {(- 2.968 [kJ/l] × O2 [l]) + (4.174 [kJ/l] × CO2 [l]) – (2.446 [kJ/g] × UN [g])} × 17.58 

(4) OXF [kJ] = {(1.719[kJ/l] × O2 [l]) + (1.719 [kJ/l] × CO2 [l]) – (1.963 [kJ/g] × UN [g])} × 39.76 

### 3.4. Statistical Analysis

Normality of residuals was tested using Shapiro-Wilks test. Both repeated measurements of oxidations) were analyzed using the following linear mixed model: Yijk = µ + αi + βij +γk +(αγ)ik + eijk . Where µ was the population mean, αi was the fixed effect of the blood sampling time (-1H, 48H, 72H and +2H), βij was a random variable corresponding to animal j in the time i. γk was the fixed effect of gender, (αγ)ik was the interactions between fixed effects. eijk was the residual error. If the systematic interaction effects did not reach significance (P < 0.05), it was eliminated from the model. Based on likelihood ratio test, the covariance structure of the repeated measurements was modelled as compound symmetry (CS), auto-regressive order 1 (AR1) or unstructured (UN) (14). All statistical analyses were performed using SAS (version 8.2). Relative standard deviation (RSD) is presented unless otherwise mentioned. Comparisons with P < 0.01 are declared highly significant, P < 0.05 significant and 0.05 < P < 0.10 are considered as trends. Pearson’s correlation coefficients were used to determine the correlations between leptin and other blood variables and between FEE, OXF, OXCHO and OXP contribution in FEE and plasma concentrations of metabolites and hormones.

## 4. Results

Changes in plasma hormones and metabolites concentrations in response to fasting and re-feeding in growing lambs are shown in Table 1. Plasma concentrations of leptin, insulin and glucose were significantly decreased by 53%, 89% and 24% within 48 hours of fasting respectively. Plasma level of leptin remained constant during the last day of fasting and did not show re-bound within two hours after re-feeding. In contrast, plasma concentration of glucose gradually increased by 72H in spite of extended fasting. The blood glucose was the only blood variable that rebounded within two hours after re-feeding. A rise in plasma insulin concentration was observed after re-feeding in spite of a sharp decrease (89%) within 48H. However, plasma concentration of insulin was still 71% lower than its concentration in fed state two hours after re-feeding. The ratio of glucose to insulin concentrations and the ratio of leptin to insulin concentrations were the highest at 48H. Surprisingly these ratios at 72H were in similar range compared to those in fed states.

**Table 1:** Changes in Plasma Metabolites and Hormones Concentrations One Hour Before (-1H) and 48 and 72 Hours After Fasting (48H and 72H) and Two Hours After Re-Feeding (+2H) in Six-Month-Old Growing Lambs

	Fasting Time				Gender		*P* values b		
	-1H	48H	72H	+2H	Female	Males	RSD	Time	Sex
**Leptin, ng/mL**	1.9 a	0.9 a	0.8 a	0.8 a	1.2	1.0	0.07	< 0.0001	0.07
**Glucose, mmol/l**	4.2 a	3.2^a^	3.7 a	4.0 a	3.8	3.8	0.07	< 0.001	NS
**Insulin, ng/mL**	0.38 a	0.04 b	0.21 a	0.11 a	0.16	0.21	0.03	< 0.0001	NS
**Glucose/insulin**	14 a	88 a	27 a	46 a	50	38	7.0	0.01	0.06
**Leptin/insulin**	6.3 a	25.1 a	6.0 a	9.1 a	13.7	9.6	1.8	< 0.001	0.06
**NEFA, mmol/l**	0.05 b	1.3 a	1.7 a	0.98 a	1.0	1.0	0.06	< 0.0001	NS
**BOHB, mmol/L**	0.73 a	0.69 a	0.68 a	0.56 a	0.69	0.64	0.03	< 0.0001	NS
**Urea, mmol/L**	6.9 a	11.7 a	9.5 a	9.6 a	9.3	9.5	0.31	< 0.0001	NS
**IGF-1, ng/mL**	48.7 a	19.5 a	12.7 a	12.4 a	19.4	27.3	2.5	< 0.0001	< 0.01

^a^Mean values within a row with unlike superscript letters were significantly different (P < 0.05).

^b^Values are means ± relative standard deviation (RSD)

Abbreviations: NEFA, Non-esterified fatty acids; BOHB, 3-β-hydroxybutyrate; IGF-1, Insulin like growth factor-1; NS, non-significant (P > 0.01).

Plasma concentration of NEFA increased significantly due to fasting with the highest level observed at 72H (more than 17 times of the fed state values). NEFA did not rebound to fed-state values within two hours after re-feeding. Plasma BOHB concentration did not change after fasting. However, two hours after re-feeding, plasma BOHB concentration significantly decreased compared to -1H, 48H and 72H values.

Urea concentrations in plasma rose up by 70% in response to fasting reaching the highest level by 48H. Urea concentration in plasma decreased with fasting prolongation at 72H and did not show any response to re-feeding.

IGF-I concentration in plasma decreased significantly by 60% and 67% within 48H and 72H respectively. IGF-1 concentration, similar to leptin, did not rebound to its fed-state values by two hours after re-feeding. Male lambs had significantly higher concentrations of IGF-I in plasma than female lambs (27.3 vs. 19.4 ng/mL). Females tend to have higher plasma concentrations of leptin (1.2 vs. 1.0 ng/mL P = 0.07) than males (Table 1).

Pearson’s correlation coefficients between plasma metabolite and circulating hormones concentrations in six-month old growing lambs (all times n=72) are shown in Table 2. Both Leptin and IGF-1 showed a significant positive relationship with each other, glucose and BOHB, but negative relationship with NEFA and Urea concentrations in blood. Insulin had positive relation with urea and negative relation with glucose in plasma.

**Table 2:** Pearson's Correlation Coefficients Between Plasma Metabolite and Circulating Hormones Concentrations in Six-Month-Old Growing Lambs (All Times n=72)

	IGF-1	Glucose	Insulin	Urea	NEFA	BOHB
**Leptin**	0.64 b	0.47 b	-0.18	-0.58 b	-0.71 b	0.26 a
**IGF-1**		0.31 b	-0.06	-0.48 b	-0.67 b	0.28 a
**Glucose**			-0.50 b	-0.60 b	-0.51 b	0.01
**Insulin**				0.45 b	0.22	0.01
**Urea**					0.53 b	-0.29 a
**NEFA**						-0.18

^a^ P< 0.05

^b^ P<0.01

Abbreviations: IGF-1, Insulin Like Growth Factor-1; BOHB, 3-β-hydroxybutyrate; NEFA, Non-esterified fatty acids

Fasting energy expenditure (FEE) and contribution of oxidized carbohydrate, fat and protein to FEE in females and males lambs are shown in Table 3. Males had significantly higher body weights and daily energy expenditure. RQ values and fasting energy expenditure per metabolic body weight (BW0.75) were in the same range for male and female lambs. After 72 hours of fasting, carbohydrate, fat and proteins contributeed 17% , 61% and 22 % in FEE respectively (Table 3). Pearson’s correlations of FEE and contribution of oxidized carbohydrate, fat and protein to FEE with plasma metabolites and hormones after 3 days fasting are shown in Table 4. FEE was negatively correlated to insulin and NEFA concentrations in plasma. Protein oxidation was positively correlated to insulin concentration in blood (Table 4).

**Table 3:** Fasting Body Weight, Fasting Energy Expenditure (FEE) and Contribution of Oxidized Carbohydrate, Fat and Protein to FEE in Six-Month-Old Growing Lambs

	Females (n=9)	Males (n=9)	*RSD*	*P* value
**Fasting body weight, kg**	27.3	33.5^a^	1.5	< 0.01
**Daily energy expenditure, MJ/day**	3.78	4.26^a^	0.11	< 0.01
**RQ (CO2 production/O2 consumption)**	0.771	0.765	0.007	NS
**Fasting energy expenditure, kJ/kg0.75**	321	309	8.8	NS
**Source of Energy Expenditure **				
Carbohydrate oxidation, kJ/day	0.63	0.66	0.07	NS
Fat oxidation, kJ/day	2.34	2.62	0.12	NS
Protein oxidation, kJ/day	0.83	0.98	0.07	NS
**Source of Energy Expenditure, %**				
Carbohydrate oxidation	17	15	2.2	NS
Fat oxidation	61	62	2.1	NS
Protein oxidation	22	23	1.6	NS

^a^Values are means ± relative standard deviation (RSD)

Abbreviations: RQ, Respiratory quotient; NS, Non-significant (P>0.05)

**Table 4:** Pearson’s Correlations Between Fasing Body Weight, Fasting Energy Expenditure and Contribution of Oxidized Carbohydrate, Fat and Protein to FEE and Plasma Metabolites and Hormones in Growing Lambs

	Fasting Energy Expenditure, kg0.75	RQ (CO_2_ /O_2_)	Carbohydrate Oxidation, %	Fat Oxidation , %	Protein Oxidation, %
**Fasting body weight**	-0.72 c	-0.45 a	-0.57 b	0.05	0.69 c
**Glucose**	0.02	0.03	-0.03	-0.27	0.38
**Insulin**	-0.51 b	-0.39	-0.45 a	0.06	0.53 b
**BOHB**	0.09	-0.05	-0.07	-0.04	0.15
**NEFA**	-0.67 c	-0.27	-0.34	0.01	0.45 a
**Urea**	0.01	0.25	0.27	-0.04	-0.31
**Leptin**	-0.10	-0.20	-0.22	0.05	0.22
**IGF-1**	0.21	-0.07	-0.02	0.27	-0.32

^a^ P < 0.10

^b^ P < 0.05

^c^ P < 0.01

Abbreviations: RQ, Respiratory quotient; BOHB, 3-β-hydroxybutyrate; NEFA, Non-esterified fatty acids; IGF-1, Insulin like growth factor-1

## 5. Discussion

In the present study, within 48 hours of fasting, plasma concentration of glucose was reduced and consequently release of insulin and IGF-1 was suppressed. The decrease in insulin and IGF-1 resulted in an enhanced lipolytic response and an elevation of plasma NEFA concentrations. On the other hand, plasma concentration of urea increased most probably due to use of amino acids in gluconeogenesis pathway (15). Similar results have previously been reported in humans (16-18), rodents (19) and ruminants (20-24).

The plasma leptin concentrations were decreased within 48 hours of fasting. In ruminant, plasma leptin concentrations reduce with short term fasting (1, 20, 25, 26). Similarly, in lean humans, fasting resulted in reduction of plasma concentrations of leptin (17, 18, 27). In monogastrics, fasting-induced reduction in leptin concentrations is believed to be due to increased sympathetic activity, increased ketones and free fatty acids, and/or a reduction in insulin and glucose concentrations (5, 28). In the present study, the positive correlation of leptin with glucose and IGF-1 and BOHB and negative correlation with NEFA and urea suggest that these plasma variables might act as leptin secretagogues and inhibitors in growing lambs. Chelikani et al. reported that leptin secretagogues might differ in different physiological state (21). For example, IGF-1 and glucose were the strongest predictors of plasma leptin concentrations in lactating dairy cows, but in non-lactating pregnant cows and heifers, IGF-1 and insulin contributed significantly to the variation in plasma leptin concentrations. They concluded that changes in insulin occurred before significant alterations in glucose, IGF-1, or NEFA, indicating that insulin might act as an early signal for leptin regulation in cattle (21).

Interestingly, in present study, no correlation was found between leptin and insulin. On the other hand, glucose was the strongest predictor of plasma leptin concentration. The fasting-induced increase in plasma NEFA and urea, and the negative correlation between NEFA and leptin provided evidence that the fasting-induced lipolysis subsequently increases intracellular free fatty acids that might act to down-regulate leptin gene expression, as demonstrated in rats (29, 30). In humans, insulin in combination with glucocorticoids seems to contribute to declines in leptin concentrations during fasting (28) but reported results on the role of insulin in regulating leptin are contradictory. On the other hand, a nutrient-sensing system has also been suggested that up-regulated leptin expression during hyperinsulinemia, hypertriglyceridemia and in states of insulin resistance (31). It suggests that predictors of leptin expression under insulin resistance status might differ, however to draw a conclusion, further investigation is needed.

In the present study, plasma concentration of BOHB apparently remained constant in fed and fasted state, a finding that is contrary to increased hepatic release of ketone bodies in fasting ewes (Hemitmann (1986). In ruminant animals, plasma BOHB depends on nutritional state. In fed animals, BOHB originated mainly from butyrate metabolism in the ruminal epithelium and liver upon absorption (32-34). Whereas in fasting state, liver is a net producer of ketone bodies, including BOHB, in ketogenesis processes, i.e. incomplete oxidation of NEFA (35) and no BOHB comes from ruminal epithelium. In fasting state, liver extracts NEFA from the blood in proportion to the hepatic supply (36), and it is directly related to rate of NEFA mobilization from adipose tissue depots. Increased hepatic uptake of NEFA in response to increasing plasma concentrations of NEFA is associated with a proportional increase in hepatic NEFA uptake as well as increased hepatic ketone body formation (including BOHB) (35, 37). Hence, in the three-day fasted lamb, the plasma BOHB is almost totally related to plasma NEFA concentration and rates of hepatic ketogenesis, whereas the contribution from absorption of butyrate from the ruminal epithelium is insignificant. It means that fasting increases BOHB production and re-feeding causes a quick decrease in BOHB production by the liver.

Present study showed that fasted animal has two different strategies to cope with extended fasting condition: firstly, development of an insulin resistance; and secondly, reducing energy expenditure through a switch from carbohydrate to fat metabolism. An elevation in plasma concentration of insulin was observed when fasting was prolonged from 48H to 72H. This might be due to development of insulin resistance. Development of insulin resistance basically occurs in insulin-dependent tissues thus spare glucose for metabolism in non-insulin dependent organs. On the other hand, insulin-dependent tissues are forced to shift away from glucose towards oxidation of fatty acids and ketone bodies. Thus, NEFA concentrations were increased with extended fasting, while glucose, insulin and urea concentrations in plasma tend to re-bound to the fed state values. Another strategy to cope with extended fasting is reducing energy expenditure through a switch from carbohydrate- to fat-based metabolism consequently lowering the body energy expenditure. In the present study, negative correlation of FEE with plasma concentration of NEFA indicated that fat oxidation is superior to carbohydrate oxidation during acute fasting. This strategy is reflected both in RQ values and in percentage of fat, protein and carbohydrate oxidation contribution to FEE with 61%, 22% and 17 % respectively.

Plasma concentrations of insulin, IGF-1, leptin and glucose decrease and NEFA and urea in plasma elevate within 48 hours of fasting. None of the energy status related hormones were quick to re-bond to fed-state values within two hours after re-feeding. Plasma glucose and NEFA seem to be stronger than insulin in regulating leptin production in fasting lambs. Under extended fasting condition, firstly, an insulin resistance develops presumably to spare glucose for vital organs, secondly, a fall in FEE through a switch from carbohydrate to fat-based metabolism occurs that is reflected in negative correlation between fasting energy expenditure and plasma concentration of NEFA.
